# Multi-Objective Feature Selection for Intrusion Detection Systems: A Comparative Analysis of Bio-Inspired Optimization Algorithms

**DOI:** 10.3390/s25196099

**Published:** 2025-10-03

**Authors:** Anıl Sezgin, Mustafa Ulaş, Aytuğ Boyacı

**Affiliations:** 1 Research and Development, Siemens A.S., Istanbul 34870, Turkey; 2 Department of Computer Engineering, Istanbul Arel University, Istanbul 34537, Turkey; 3 Department of Artificial Intelligence and Data Engineering, Fırat University, Elazig 23050, Turkey; mustafaulas@firat.edu.tr; 4 Department of Computer Engineering, Air Force Academy, National Defence University, Istanbul 34149, Turkey; aytug.boyaci@msu.edu.tr

**Keywords:** intrusion detection systems, multi-objective optimization, feature selection, bio-inspired algorithms, IoT security, network security

## Abstract

The increasing sophistication of cyberattacks makes Intrusion Detection Systems (IDSs) essential, yet the high dimensionality of modern network traffic hinders accuracy and efficiency. We conduct a comparative study of multi-objective feature selection for IDS using four bio-inspired metaheuristics—Grey Wolf Optimizer (GWO), Genetic Algorithm (GA), Particle Swarm Optimization (PSO), and Ant Colony Optimization (ACO)—on the X-IIoTID dataset. GA achieved the highest accuracy (99.60%) with the lowest FPR (0.39%) using 34 features. GWO offered the best accuracy–subset balance, reaching 99.50% accuracy with 22 features (65.08% reduction) within 0.10 percentage points of GA while using ~35% fewer features. PSO delivered competitive performance with 99.58% accuracy, 32 features (49.21% reduction), FPR 0.40%, and FNR 0.44%. ACO was the fastest (total training time 3001 s) and produced the smallest subset (7 features; 88.89% reduction), at an accuracy of 97.65% (FPR 2.30%, FNR 2.40%). These results delineate clear trade-off regions of high accuracy (GA/PSO/GWO), balanced (GWO), and efficiency-oriented (ACO) and underscore that algorithm choice should align with deployment constraints (e.g., edge vs. enterprise vs. cloud). We selected this quartet because it spans distinct search paradigms (hierarchical hunting, evolutionary recombination, social swarming, pheromone-guided foraging) commonly used in IDS feature selection, aiming for a representative, reproducible comparison rather than exhaustiveness; extending to additional bio-inspired and hybrid methods is left for future work.

## 1. Introduction

The ever-growing footprint of Internet of Things (IoT) and Industrial IoT (IIoT) deployments has fundamentally reshaped contemporary cyber-risk. Pervasive sensing, continuous connectivity, and heterogeneous device ecosystems expand the attack surface while compressing defenders’ reaction windows. In this context, Intrusion Detection Systems (IDSs) have become a central control point—able to surface deviations from expected behavior, support triage, and provide early warning before an incident escalates into service degradation or lateral movement [1]. At the same time, the telemetry on which modern IDS operate is increasingly high-dimensional: packet headers and flows, protocol and timing statistics, and contextual features accumulate rapidly in both rate and variety, amplifying the classical curse of dimensionality. Redundant or weakly informative variables can erode generalization, inflate both training and inference costs, and cause decision latency that undermines real-time defense, especially in bandwidth- and energy-constrained IoT/IIoT settings [1,2]. In operational networks, these issues are compounded by non-stationarity (concept drift), device heterogeneity, and imbalanced class distributions typical of rare but critical attack events.

Against this backdrop, feature selection is not merely a pre-processing convenience; it is a first-class design element in IDS pipelines. Properly chosen subsets can sharpen class boundaries, reduce overfitting, and lower compute and memory footprints without sacrificing discriminative power. However, feature selection in IDS is inherently multi-objective: practitioners seek high detection performance while minimizing subset size and controlling false positives (FPR), false negatives (FNR), and runtime/throughput under fixed resource budgets. Objectives frequently demonstrate conflict: adding features may marginally improve accuracy yet degrade latency or increase error asymmetry in ways that are unacceptable for deployment. These trade-offs motivate multi-objective optimization (MOO), which returns Pareto-optimal solution sets, enabling designers to choose operating points that best fit their constraints rather than a single “one-size-fits-all” optimum [3,4].

Bio-inspired metaheuristics are particularly attractive for IDS feature selection because they can efficiently explore vast, discrete search spaces with minimal assumptions about objective smoothness or convexity. Among them, Grey Wolf Optimizer (GWO), Genetic Algorithm (GA), Particle Swarm Optimization (PSO), and Ant Colony Optimization (ACO) are canonical choices with long, diverse usage histories in security and CPS/ICS contexts [5,6,7]. Each embodies a distinct population-based search dynamic that yields complementary exploration–exploitation behaviors: GWO leverages leader-guided encircling that often produces stable convergence; GA uses crossover/mutation to preserve diversity and probe a broad Pareto front; PSO employs velocity-driven swarming for rapid convergence toward promising regions; and ACO constructs sparse subsets through stigmergic (pheromone-guided) reinforcement that naturally favors compact representations. Selecting a representative quartet that spans hierarchy, evolution, swarm dynamics, and stigmergy allows a balanced comparison of how different search philosophies navigate IDS-specific trade-offs under a common experimental protocol. Our study adopts exactly this rationale and evaluates MOGWO, MOGA, MOPSO, and MOACO as multi-objective feature selectors within a unified framework, later showing that each method concentrates on a different region of the accuracy–subset–runtime design space.

A robust dataset is essential to ensure results reflect realistic IIoT conditions. We evaluate on X-IIoTID, a large, device- and connectivity-agnostic IIoT intrusion dataset curated for research use, comprising heterogeneous devices, protocols, and attack vectors. By design, it stresses both the dimensionality and diversity that practitioners face in the field, making it a suitable benchmark for assessing the efficacy and efficiency of multi-objective feature selection in IDS. In our pipeline, the dataset undergoes standardized cleaning, encoding, scaling, and stratified splits to avoid distributional artifacts; then, each optimizer produces feature subsets that are assessed by the same base classifier and metrics to enable fair, apples-to-apples comparisons. This design choice is deliberate: many earlier IDS works use disparate datasets or single-objective criteria, hindering cross-paper comparability; our protocol minimizes such confounders and foregrounds the true algorithmic trade-offs [1,8].

Despite the breadth of IDS literature, there remains a gap in controlled, multi-objective comparisons of canonical bio-inspired methods on modern IIoT traffic. Deep learning and ensemble approaches have advanced state of the art in many settings, but they often either (i) avoid explicit multi-objective selection, (ii) rely on legacy benchmarks, or (iii) adopt non-uniform pre-processing and evaluation protocols that complicate head-to-head assessment [1,6,7,9]. By contrast, we structure our evaluation to isolate the contribution of the feature selection strategy itself. Concretely, we (a) fix the downstream classifier and metrics across all runs, (b) execute 30 independent trials per algorithm to average out stochasticity, and (c) report a comprehensive metrics suite spanning accuracy, FPR, FNR, selected-feature counts/reduction ratios, runtime, and multi-objective indicators such as Pareto diversity.

Our aim is therefore twofold. First, we seek to standardize an end-to-end, reproducible evaluation of four widely used bio-inspired multi-objective feature selection algorithms for IDS on X-IIoTID. Second, we map the resulting trade-off regions to practical deployment contexts: when maximum precision and minimal FPR dominate (e.g., enterprise networks with powerful backends), MOGA is favorable; when accuracy must be high yet model size and retraining cost should remain moderate (e.g., balanced corporate settings), MOGWO stands out; when resource budgets are tight and retraining speed matters (e.g., edge/IoT devices), MOACO offers compelling sparsity and training speed; and when a service can absorb heavier compute for marginal gains (e.g., cloud batch environments), MOPSO provides competitive accuracy. These qualitative descriptors are backed by the quantitative results summarized in our findings.

This paper makes the following contributions:

1. We formulate IDS feature selection as a bi-objective problem (classification error and subset size) and implement MOGWO, MOGA, MOPSO, and MOACO within a single, unified evaluation pipeline that fixes pre-processing, classifier choice, metrics, and repetition counts for fair comparison.
2. We provide a quantitative, Pareto-aware comparison on X-IIoTID, reporting accuracy, FPR, FNR, runtime, and subset size/reduction, alongside indicators of Pareto-front diversity, so that designers can reason about accuracy–efficiency–latency compromises rather than a solitary score.
3. We translate empirical results into actionable guidance: GA for maximum accuracy and lowest FPR; GWO for the best accuracy–subset balance (near-best accuracy with substantially fewer features); PSO for near-best accuracy albeit at higher training time; and ACO for the fastest training and most aggressive sparsity, all under the same protocol. The consolidated outcomes demonstrate these distinctions clearly.
4. We delineate a representative scope of bio-inspired optimization—hierarchy (GWO), evolution (GA), swarm (PSO), and stigmergy (ACO)—as a reproducible baseline for IDS feature selection. While not exhaustive (e.g., DE, ABC, FA, CS, BA, Whale Optimization remain of interest), this scope meaningfully spans major search paradigms and operational footprints; extending the comparison to a broader family is left to future work.

In this study we optimize two objectives (i) maximize classification accuracy, (ii) minimize the number of selected features, and we evaluate using a fixed metric set: Accuracy, False Positive Rate (FPR), False Negative Rate (FNR), Selected Features (also reported as % reduction), and Training Time. We use this metric set consistently throughout the paper for clarity and comparability.

This paper is structured to provide a comprehensive overview of our research methodology, findings, and their implications. Following this introduction, Section 2 provides a detailed review of existing literature on intrusion detection, feature selection, and bio-inspired algorithms across various network types, including IoT, IIoT, and vehicular networks. Section 3 outlines the problem formulation, dataset preparation using the X-IIoTID dataset, and the specific implementation details of the four multi-objective algorithms and our evaluation framework. The core findings, including the comparative performance analysis and trade-off discussions, are presented in Section 4. Section 5 summarizes the key takeaways, provides practical recommendations for deployment, and suggests avenues for future research.

## 2. Related Work

### 2.1. IDS for IoT/IIoT and Sensor-Centric Networks

IoT/IIoT deployments have pushed intrusion detection toward compact, feature-efficient pipelines that can operate under tight memory, power, and latency constraints. Automation-oriented frameworks streamline preprocessing, feature selection, and model selection to reduce engineering effort while maintaining high accuracy in industrial settings [10,11]. In Wireless Sensor Networks, integrated designs combine sequence models for detection with response policies and bespoke optimizers for feature selection, and then adapt these components to general IoT scenarios with similarly strong performance [12,13]. Orthogonally, privacy-preserving learning has matured: federated variants enable real-time, lightweight DDoS detection on distributed nodes without centralizing raw data [14]. Dimensionality-reduction-centric pipelines (e.g., stacked autoencoders with gradient-boosted trees) demonstrate that careful compression can retain accuracy while lowering runtime footprints [15], and attention-fusion classifiers report gains on zero-day detection with improved explainability [16]. Optimization-assisted tuning (e.g., GS-PSO for RPL-IoT) boosts Random Forest efficacy but usually treats feature selection implicitly and under single-objective criteria [17]. More recent efforts address practical difficulties such as imbalance and concept drift in IoT streams [2]. Complementary lines convert traffic to image representations for deep classifiers [18], while surveys synthesize privacy and security constraints for IIoT at cloud/edge/fog boundaries [19]. Collectively, these works advance accuracy, automation, and privacy but seldom expose a standardized accuracy–subset–runtime trade-off within a multi-objective protocol.

### 2.2. Intrusion Detection in Vehicular Networks (VANET/IoV)

Vehicular environments demand lightweight spatio-temporal modeling and strict latency guarantees. Compact ConvGRU architectures combine shallow CNNs with recurrent units to capture traffic dynamics efficiently and deliver deployable detectors [20]. Feature selection is sometimes entwined with custom ranking schemes and fuzzy deep ensembles to raise accuracy [21], or incorporated into multi-stage, cloud-assisted frameworks designed for low footprint [22]. Federated variants further enable collaborative learning while preserving privacy in distributed vehicular settings [23], and surveys consolidate ensemble patterns that underpin many current IoV IDS designs [24]. Despite progress, feature selection is rarely posed as an explicit multi-objective optimization layer; reporting on subset compactness, runtime, and Pareto diversity remains limited and non-uniform across studies.

### 2.3. SDN, Cloud, and Big-Data Environments

For SDN, contrastive-learning pipelines with two-tier classifiers—sequence models in the first stage followed by convolutional–bidirectional layers—achieve robust DDoS detection under dynamic traffic [25]. Ensemble feature selection coupled with MLPs targets variable-rate DDoS scenarios with improved detection [26]. A broader review organizes advances by target networks (Cloud, IoT, SDN) and surfaces recurring challenges in training-data quality and feature-selection practice [1]. However, standardized apples-to-apples evaluations that make multi-objective trade-offs explicit are still uncommon in these environments.

### 2.4. Other Domains and General ML/DL Directions

Beyond the above settings, privacy-centric SCADA frameworks combine feature optimization (e.g., SHAP, RFE, PCA) with federated/homomorphic techniques and active defenses (honeypots, firewalls) to strengthen protection of critical infrastructure [27], while CPS-tailored IDS proposals address domain-specific constraints such as tight control loops and safety tolerances [28]. Hybrid pipelines pair classical learners with nature-inspired optimizers for malware/IDS tasks and report accuracy gains [29]. Deep architectures that marry PCA with Transformer-style models improve representation quality for classification [9], and graph-learning pipelines integrate nature-inspired selection with attention-based GCNs [5]. Industrial settings leverage GWO-assisted selection with autoencoding for anomaly detection [3], and hybrid PSO–Firefly searches target high-dimensional spaces effectively [4]. In cloud contexts, H2RNN combines SVM-RFE, PCA, and PSO for hybrid feature selection with recurrent–associative classification [30]; other deep approaches emphasize transfer learning and explainable AI to increase adaptability and transparency [31,32]. Additional advances include attention-enhanced GhostNets optimized by SMO for DDoS [33], tensor-based large-scale detectors [34], lightweight DCNNs for constrained devices [6], metric-learning with dual one-class units [35], and ensemble pipelines with PSO-tuned forests [7]. Despite breadth, most studies remain single-objective and rarely adopt a uniform protocol that jointly reports accuracy, subset size, and runtime.

### 2.5. Automation, RL/LLMs, and Privacy-Preserving Learning

Automation-driven approaches use GA-guided neural architecture search to optimize CNNs for IoT intrusion detection, reducing manual design effort [36]. LLM-augmented systems integrate Retrieval-Augmented Generation to improve adaptation to unknown attacks [37]. Federated-learning variants span IoMT, IoT, and IoHT: lightweight XGBoost with Bayesian tuning of local learners, knowledge-distillation with proximal regularization for heterogeneous devices, BERT-based encoders with efficient update rules, and ε-differential privacy for sensitive data [38,39,40,41]. Unsupervised, privacy-preserving detection of novel DGA malware leverages VAE-based latent representations with resource-aware client selection, avoiding raw-data sharing [42]. These directions advance privacy and automation, yet generally do not cast feature selection as a multi-objective problem with standardized reporting on runtime and subset compactness.

### 2.6. Comparison, Gaps, and Contributions

In IoT/IIoT and WSN settings, automation- and lightweight-centric pipelines improve practicality (AutoML; dimensionality reduction; attention/fusion), and privacy-preserving variants via FL enable distributed detection, yet evaluations remain mostly single-objective with non-uniform reporting on subset compactness and runtime [10,11,12,13,14,15,16,17,18,19]. In vehicular networks, compact spatio-temporal models (e.g., ConvGRU) and customized feature-ranking or fuzzy ensembles push accuracy and enable cloud-assisted or federated deployment, but feature selection is rarely treated as an explicit multi-objective layer and Pareto diversity is seldom analyzed [20,21,22,23,24]. In SDN/cloud environments, contrastive-learning pipelines and ensemble feature selection improve DDoS detection, while surveys catalog cross-domain challenges; still, apples-to-apples multi-objective baselines are uncommon [1,25,26]. Across general ML/DL and nature-inspired lines—ranging from GWO/PSO-based selection to PCA-Transformer, graph-attention variants, and TL/XAI ensembles—accuracy and robustness gains are reported, yet standardized reporting of the accuracy–subset–runtime compromise is rare and protocols vary widely [3,4,5,6,7,9,29,30,31,32,33,34,35]. Finally, automation, RL/LLMs, and FL-privacy directions (GA-guided NAS, RAG + LLM, FL with XGBoost/BERT/KD and ε-DP, VAE-based unsupervised DGA detection) advance adaptability and privacy but typically keep feature selection implicit rather than framing it as a multi-objective optimization with reproducible, deployment-oriented metrics [36,37,38,39,40,41,42].

From this synthesis, five gaps consistently emerge. (1) Single-objective bias: most evaluations optimize or report a single criterion, leaving the fundamental trade-off among accuracy, feature-subset size, and runtime implicit rather than explicit. (2) Non-uniform baselines: datasets, preprocessing, base classifiers, metrics, and repetition counts vary widely, undermining apples-to-apples comparison across papers. (3) Incomplete deployment metrics: training time and explicit feature-reduction ratios are often missing or only partially reported, obscuring real-world feasibility on edge or high-throughput systems. (4) Limited Pareto awareness: few studies visualize or analyze Pareto fronts to expose alternative operating points for edge, enterprise, and cloud constraints. (5) Underused bio-inspired MOO framing: even when nature-inspired search is employed, it is commonly embedded as a tuning heuristic rather than posed as a multi-objective feature-selection problem with standardized, reproducible reporting.

We address these gaps by casting IDS feature selection as a bi-objective problem and evaluating MOGWO, MOGA, MOPSO, and MOACO under a single, standardized pipeline on X-IIoTID. Preprocessing, the base classifier, metrics, and repetition counts are fixed to ensure fair, reproducible comparisons. We report a complete metric suite—accuracy, FPR, FNR, selected-feature counts/reduction, and total training time—so that the accuracy–subset–runtime compromise is explicit rather than implied. Results are presented in a Pareto-aware manner, delineating distinct operating regions (high-accuracy, balanced, and efficiency-oriented) and yielding practical guidance for mapping algorithm choice to deployment constraints (edge vs. enterprise vs. cloud).

## 3. Methodology

### 3.1. Problem Formulation and Dataset Preparation

The challenge of multi-objective feature selection within the scope of intrusion detection systems is a highly compound optimization challenge that requires careful mathematical formulation and a structured plan of dataset preparation. In our study, the feature selection challenge has been framed as a bi-objective optimization challenge where the intention is to simultaneously optimize two rival objectives and maintain the integrity and representativeness of the primary data.

The mathematical formulation of our multi-objective feature selection problem can be expressed as follows. Let *X* be defined as in (1) represent the complete feature set containing *n* features, and let *S* ⊆ *X* represent a selected subset of features.

(1) $$X=\{x_{1},x_{2},\ldots ,x_{n}\}$$

The optimization problem seeks to find the optimal subset *S* that minimizes two objective functions: the classification error, as shown in (2), and the feature reduction ratio, as given in (3). The problem is subject to the constraint given in (4), where each element indicates whether a feature is selected (1) or not (0). We cast feature selection as a bi-objective problem: (i) maximize classification accuracy on the validation fold, and (ii) minimize the number of selected features used by the downstream classifier. Equivalently, the minimization form is given in Equation (2); here, the constant “1” denotes the unit upper bound of normalized accuracy (100%), not the accuracy with the full feature set *X*.

(2) $$f_{1}\left(S\right)=1-Accuracy\left(S\right)$$

(3) $$f_{2}\left(S\right)=\frac{\left|S\right|}{\left|X\right|}$$

(4) $$S\in \{0,1{\}}^{n}$$

Here, *X* is the full feature set and *S* ⊆ *X* is the selected subset; ∣⋅∣ denotes cardinality; Accuracy (*S*) ∈ [0, 1] is the validation accuracy for subset *S*. The constant “1” denotes the unit upper bound of normalized accuracy (100%), not the accuracy obtained with the full feature set *X*. We use 1 − Accuracy (*S*) so that both objectives are minimized on a comparable scale.

This formulation captures the fundamental trade-off in feature selection where improving classification accuracy often requires including more features, thereby increasing computational complexity and potential overfitting. The Pareto optimality concept, as defined in (5), becomes crucial in this context, as it allows us to identify multiple non-dominated solutions that represent different balance points between these conflicting objectives.

(5) $$\neg \exists S\prime :(\forall if_{i}(S\prime )\leq f_{i}(S^{*}))\wedge (\exists jf_{j}(S\prime )<f_{j}(S^{*}))$$

The X-IIoTID is the main assessment criterion of the current study. The dataset is a very large dataset of network traffic data that has been especially crafted to reflect the novel nature and security issues of current IoT infrastructure. The dataset consists of network traffic that has been gathered through a realistic testbed of an IoT with various types of devices like sensors, actuators, smart home devices, and industrial control systems.

We evaluate on the X-IIoTID dataset, which aggregates IIoT traffic across heterogeneous devices, protocols, and attack types. To support reproducibility and a fair, apples-to-apples comparison across optimizers, we apply a uniform preprocessing pipeline (cleaning, encoding, scaling) and a stratified split. Table 1 consolidates the core characteristics: size, feature taxonomy, class distribution, missingness, preprocessing, and data splits. In this study, the IDS is instantiated as a binary anomaly detector—i.e., it distinguishes normal versus attack traffic. We adopt the binary task to isolate the effect of multi-objective feature selection across optimizers under a fixed classifier, avoiding confounds from class imbalance and label granularity that arise in per-attack multi-class setups. The hierarchical attack labels available in X-IIoTID are retained for future work extending the pipeline to family/sub-type classification.

The dataset consists of various attack scenarios, such as Distributed Denial of Service (DDoS) attacks, Man-in-the-Middle attacks, data injection attacks, and several malware infections that aim at targeting Internet of Things (IoT) devices solely. All the network flows in the dataset are described by numerous features that are derived from the header of the packets, flow statistics, and behavioral profiles. These features range from simple network features such as source and destination IP addresses, port addresses, protocol types, sizes of packets, and durations of flows, to more sophisticated statistical features such as durations of packet inter-arrivals, variations in flow rates, and protocol-specific features.

The preprocessing pipeline developed for the X-IIoTID dataset includes several indispensable procedures dedicated to ensuring data integrity and compatibility with our optimization algorithms. We start by performing a thorough cleansing of the data to remove superfluous metadata columns such as timestamps and those identifiers that do not feature directly in the intrusion detection task at hand. We then treat missing values by utilizing a combination of statistical imputations and domain knowledge, thus guaranteeing that the imputed observations do not impart any bias to the classification methodology adopted.

In the dataset, categorical variables are converted by the application of appropriate techniques, such as one-hot encoding of nominal variables and ordinal encoding of ordinal variables. This encoding ensures that all the features are given numerically presentable forms that remain appropriate for machine learning algorithms, with the preservation of the semantic meaning of the categorical information.

Scaling of features is a crucial preprocessing step since the features within network traffic data often show drastically different scales and distributions. We apply standardization using z-score normalization to ensure that all the features have a mean of zero and a variance of one, thus preventing the possibility of some single feature on account of scale differences dominating the optimization procedure.

The dataset is partitioned into training and testing sets using stratified sampling to maintain the original class distribution in both subsets. We allocate 80% of the data for training and 20% for testing, ensuring that the optimization algorithms have sufficient data for learning while maintaining an independent test set for unbiased performance evaluation.

### 3.2. Multi-Objective Algorithm Implementation and Evaluation Framework

Bio-inspired optimization is a broad family; beyond GWO, GA, PSO, and ACO, notable alternatives include Differential Evolution (DE), Artificial Bee Colony (ABC), Firefly Algorithm (FA), Cuckoo Search (CS), Bat Algorithm (BA), Whale Optimization, and others. We restricted the study to four canonical, mature methods that (i) have strong adoption in IDS/feature-selection literature, (ii) span diverse search mechanics (hierarchy, evolution, swarm, stigmergy), (iii) offer well-understood parameterizations for reproducibility, and (iv) cover continuous and discrete subset search effectively. Future work will extend the comparison to additional bio-inspired and hybrid approaches under the same experimental protocol.

The implementation of multi-objective bio-inspired optimization algorithms requires careful consideration of algorithm-specific parameters, convergence criteria, and performance evaluation metrics. This section details the implementation specifics of four prominent algorithms and establishes a comprehensive evaluation framework for fair comparison.

The Multi-Objective Grey Wolf Optimizer (MOGWO) represents an adaptation of the traditional Grey Wolf Optimizer for multi-objective scenarios. The algorithm simulates the social hierarchy and hunting behavior of grey wolves, where the population is divided into four categories: alpha (α), beta (β), delta (δ), and omega (ω) wolves. The position of each wolf is updated based on the locations of the alpha, beta, and delta wolves, as defined in (6)–(9). The alpha wolves represent the best solutions found so far, beta wolves represent the second-best solutions, delta wolves represent the third-best solutions, and omega wolves represent the remaining population members.

(6) $$D_{\alpha }=\left|C_{1}\cdot X_{\alpha }-X\right|,\;X_{1}=X_{\alpha }-A_{1}\cdot D_{\alpha }$$

(7) $$D_{\beta }=\left|C_{2}\cdot X_{\beta }-X\right|,\;X_{2}=X_{\beta }-A_{2}\cdot D_{\beta }$$

(8) $$D_{\delta }=\left|C_{3}\cdot X_{\delta }-X\right|,\;X_{3}=X_{\delta }-A_{3}\cdot D_{\delta }$$

(9) $$X\left(t+1\right)=\frac{X_{1}+X_{2}+X_{3}}{3},\;A=2a\cdot r_{1}-a,\;C=2r_{2}$$

In our MOGWO code, we keep an external archive to save non-dominated solutions encountered during the course of optimization. The archive is maintained at every iteration by the use of fast non-dominated sorting and calculation of the crowding distance to maintain diversity of solutions stored. Parameters of the algo-rithm include a population of 30 individuals, 50 max iterations, and the archive size restriction of 100 solutions to avoid memory overflow and retain di-versity of solutions.

The Multi-Objective Genetic Algorithm (MOGA) implementation follows the principles of evolutionary computation, including specific modifications aimed at multi-objective optimization. The algorithm maintains a population of candidate solutions presented as binary chromosomes where every bit indicates the selection of the respective feature. The offspring are generated by uniform crossover and bit-flip mutation methods, given by Equations (10) and (11), respectively. The selection procedure follows the tournament selection that is based on the Pareto dominance and the crowding distance, allowing both the convergence on the Pareto front and the maintenance of the diversity.

(10) $$r∼U\left(0,1\right),\;\mathrm{child}\left[j\right]={\mathrm{parent}}_{1}\left[j\right]I_{\;r<0.5}+{\mathrm{parent}}_{2}\left[j\right]I_{\;r\geq 0.5}$$

(11) $$u∼U\left(0,1\right),\;S_{j}^{\prime }=\left(1-I_{\;u<p_{m}}\right)\;S_{j}+I_{\;u<p_{m}}\;\left(1-S_{j}\right)$$

Crossover operations utilize uniform crossover with a probability of 0.8, where bits are exchanged between parent chromosomes to create offspring solutions. Mutation operations employ bit-flip mutation with a probability of 0.1, randomly flipping bits in the chromosome to introduce variation and prevent premature convergence. The algorithm runs for 50 generations with a population size of 30 individuals, and elitism ensures that the best solutions from each generation are preserved.

The Multi-Objective Particle Swarm Optimization (MOPSO) algorithm adapts the concept of particle swarm intelligence for multi-objective feature selection. Each particle in the swarm represents a potential feature subset, and particles move through the solution space guided by their personal best positions and global best positions discovered by the swarm. The velocity and position of each particle are updated according to (12) and (13).

(12) $$v_{i}^{t+1}=w\;v_{i}^{t}+c_{1}\;r_{1}\;\left(pbest_{i}-x_{i}^{t}\right)+c_{2}\;r_{2}\;\left(gbest_{i}-x_{i}^{t}\right)$$

(13) $$x_{i}^{t+1}=x_{i}^{t}+v_{i}^{t+1}$$

The velocity update mechanism incorporates inertia weight (*w* = 0.9) to balance exploration and exploitation, personal acceleration coefficient (*c*_1_ = 2.0) to encourage particles to return to their previously discovered best positions, and social acceleration coefficient (*c*_2_ = 2.0) to attract particles toward globally discovered best positions. The algorithm maintains an external archive of non-dominated solutions, and particles select their guide positions from this archive using roulette wheel selection based on crowding distance.

The Multi-Objective Ant Colony Optimization (MOACO) algorithm draws inspiration from the foraging behavior of ants and their ability to find optimal paths through pheromone communication. In our implementation, artificial ants construct feature subsets by traversing a construction graph where each node represents a feature and edges represent the decision to include or exclude features. The pheromone update rule and the state transition probability are given in (14) and (15).

(14) $$\tau_{ij}\leftarrow \left(1-\rho \right)\;\tau_{ij}+\sum_{k=1}^{m}\Delta \tau_{ij}^{k}$$

(15) $$P_{ij}^{k}=\frac{\tau_{ij}^{\alpha }\;\eta_{ij}^{\beta }}{\sum_{l\in \mathrm{allowe}d_{k}}\tau_{il}^{\alpha }\;\eta_{il}^{\beta }}$$

The pheromone update mechanism incorporates multiple pheromone matrices corresponding to different objectives, allowing ants to follow different pheromone trails based on the optimization goals. The algorithm parameters include pheromone importance (α = 1.0), heuristic importance (β = 2.0), and pheromone evaporation rate (ρ = 0.1). The colony consists of 30 ants that construct solutions over 50 iterations, with pheromone updates based on the quality of discovered solutions.

To provide a consistent and fair assessment across different multi-objective heuristics, we adopt a Decision Tree Classifier as the base machine learning model of performance assessment. The choice of the Decision Tree Classifier is motivated by several factors: its computational effectiveness, making it suitable for the rapid evaluation of large feature subsets; its interpretability, which allows the study of the importance of features and selection patterns; its robustness of features’ scaling, thus eliminating eventual scaling-based bias due to preprocessing differences; and the shown effectiveness of the Decision Tree Classifier in network intrusion detection tasks by the literature.

The evaluation process follows a typical procedure throughout all the algorithms. Once the multi-objective optimization scheme presents the optimum feature subset, the corresponding features are picked from the training and test datasets. A Decision Tree Classifier is also trained on the features selected on the training set. The learned model is then evaluated on the test set with the same feature subset to obtain unbiased estimates of the performance like classification accuracy, false positive rate, and false negative rate.

The assessment methodology encompasses several different performance measures that allow one to comprehensively analyze the effectiveness of the algorithm. The assessment of classification effectiveness encompasses the accuracy, precision, recall, F1-score, false positive rate, and false negative rate. Performance of the feature selection is assessed by the number of features selected, the ratio of feature reduction, and feature selection consistency over several iterations.

The multi-objective performance measures include hypervolume that estimates the volume of the objective space that is dominated by the Pareto front; spacing that estimates the regularity of the distribution of the solutions along the Pareto front; and coverage that accounts for the dominance relations of the Pareto fronts produced by various algorithms.

The computational effectiveness is quantified through measures of execution time, memory usage, and rate of convergence. Statistical significance testing procedure applies non-parametric techniques to detect significant differences between the algorithms’ performances. The experimental setup provides 30 independent runs per algorithm to ensure statistical soundness and to overcome the randomly inherent nature of the optimization procedure.

The overall procedure of the suggested multi-objective feature selection and evaluation framework, that is, the data preprocessing, optimization, and classification performance assessment, is described by Algorithm 1.
**Algorithm 1:** Multi-Objective Feature Selection Framework Procedure
INPUT: Dataset OUTPUT: Performance metrics and visualizations // Data Preprocessing LOAD dataset. SPLIT dataset into training (80%) and testing (20%) sets. STANDARDIZE features of both sets. // Optimization and Training INITIALIZE algorithms: GWO, GA, PSO, ACO. FOR each algorithm IN [GWO, GA, PSO, ACO]:  best_features, pareto_front = OPTIMIZE(algorithm, training_set).  classifier = TRAIN_CLASSIFIER(best_features, training_set).  metrics = EVALUATE(classifier, testing_set).  STORE results (accuracy, FPR, FNR, runtime).

**Figure 1:**
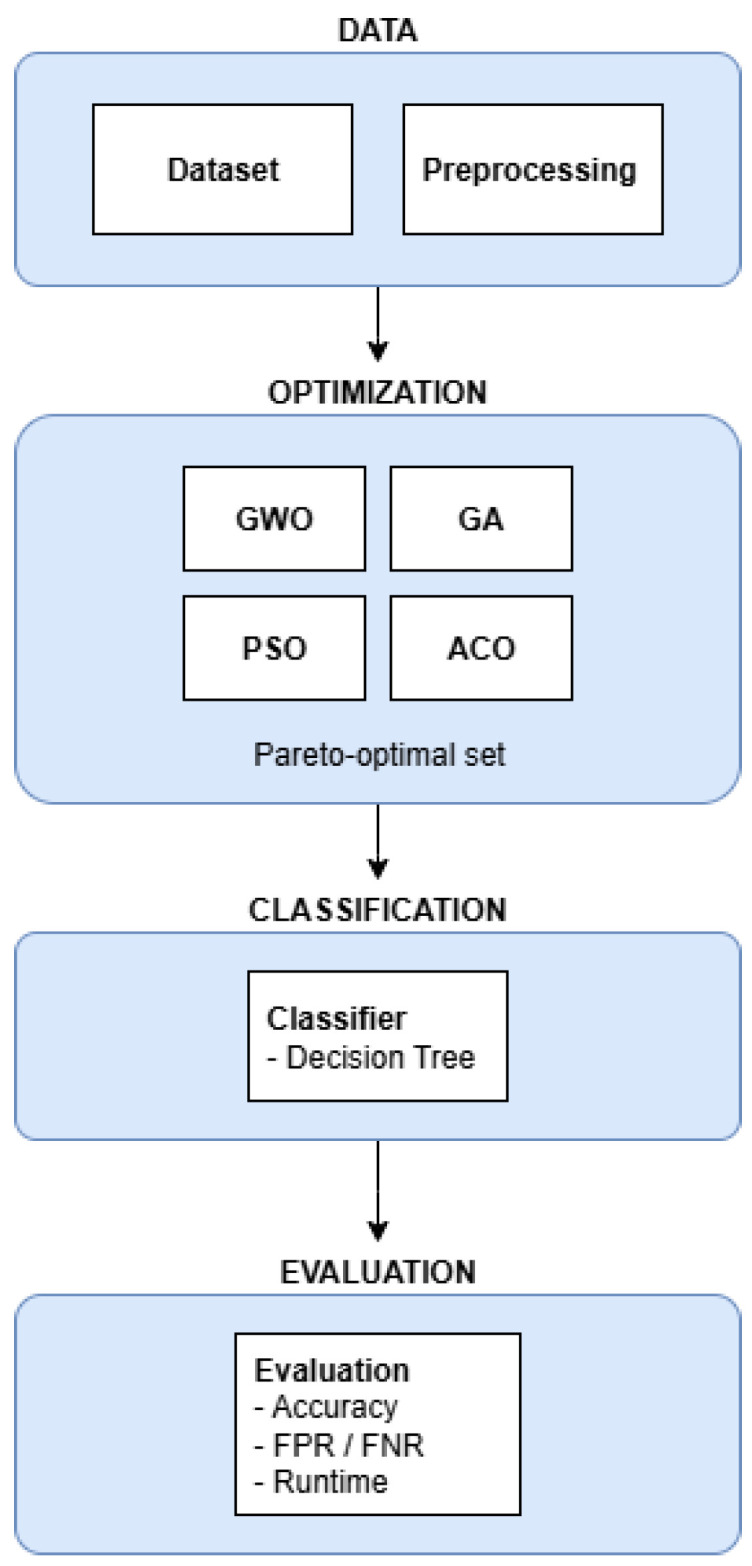
Diagram of the proposed multi-objective feature selection and evaluation framework.

The suggested multi-objective feature selection framework is outlined in Figure 1. The diagram is rigorously split into four main components: Data, Optimization, Classification, and Evaluation. The Data component handles dataset preparation and preprocessing, the Optimization component makes use of bio-inspired algorithms (GWO, GA, PSO, ACO) to generate Pareto-optimal sets of features, the Classification component deploys a Decision Tree classification scheme, and the Evaluation component estimates the performance measures like accuracy, false positive and false negative rates, and runtime. This modular structure efficiently illustrates the integration of algorithms by the overall IDS framework.

## 4. Results and Discussion

### 4.1. Comparative Performance Analysis and Algorithm Evaluation

The current section presents an in-depth analysis of the experimental results obtained by the usage of four multi-objective bio-inspired optimization techniques within the area of feature selection for intrusion detection systems. Performance evaluation spans several dimensions, including classification precision, effectiveness of feature reduction, computational complexity, and the efficacy of the multi-objective optimization procedure. All optimization algorithms and the subsequent comparative analysis were implemented in Python 3.10. To evaluate the feature subsets produced by the optimizers, we employed a Decision Tree Classifier as the downstream model. To maintain a fair comparison, we applied a consistent set of hyperparameters across methods wherever applicable: population size = 30 (interpreted as swarm size for PSO and number of ants for ACO) and maximum iterations = 50 (generation for GA). Beyond these common settings, we configured method-specific controls as follows. For Genetic Algorithm, we set crossover_rate = 0.8 and mutation_rate = 0.1. The Particle Swarm Optimization used an inertia weight *w* = 0.9 with cognitive and social coefficients c_1_ = 2.0 and c_2_ = 2.0. For Ant Colony Optimization, we used alpha = 1.0 (pheromone influence), beta = 2.0 (heuristic influence), and rho = 0.1 (evaporation rate). The Grey Wolf Optimizer followed the standard leader-guided encircling update and did not require additional method-specific hyperparameters in our implementation. To mitigate stochastic effects, each algorithm was executed for 30 independent runs, and we report the mean results; wall-clock runtime was measured per run (excluding I/O) under the same software stack. We acknowledge that MOGA and MOPSO incur long wall-clock times (≈ 7 h + per run); in practice, efficiency can be improved via lower iteration budgets, patience-based early stopping, warm-starts from prior runs, and parallel evaluation on multi-core/GPU hardware.

Table 2 summarizes the comparative performance of the four algorithms—MOGWO, MOGA, MOPSO, and MOACO—when used for the purpose of feature selection for intrusion detection. The table gives detailed descriptions of classification accuracy, number of features chosen, number of features percentage reduction, false positive rate (FPR), false negative rate (FNR), runtime, and the number of Pareto-optimal solutions produced by each of the techniques. Overall, these parameters provide an all-round evaluation that not only displays the predictive effectiveness of each of the methods, but also estimates the ease of reducing the dimensionality of the features, reducing error rates, and computational feasibility. Furthermore, the parameter of Pareto solutions presents the range of the optimum trade-offs identified by each of the methods, which is very important for balancing competing objectives within the area of multi-objective optimization scenarios.

**Figure 2:**
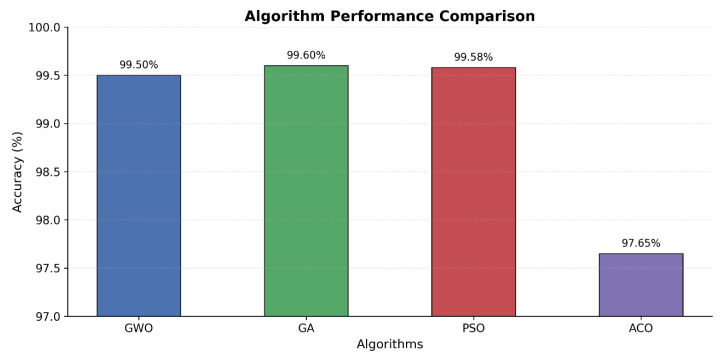
Algorithm performance comparison.

The experimental findings show distinct differences in the performance of the four multi-objective algorithms when applied to the X-IIoTID dataset consisting of 820,834 network traffic instances described by 63 features. As can be seen in Figure 2, the highest accuracy of 99.60% and the lowest false positive rate of 0.39% were achieved by the Genetic Algorithm (MOGA), albeit at the cost of 34 features and a high computational time of 25,486 s. On the contrary, the Grey Wolf Optimizer (MOGWO) showed a remarkable balance between accuracy of 99.50% and using 22 features (65.08% reduction) with a moderate computational cost of 6644 s. These results indicate that GWO attains near-best accuracy while using ~35% fewer features than GA (22 vs. 34), clarifying our use of the phrase “best balance between accuracy and features”.

**Figure 3:**
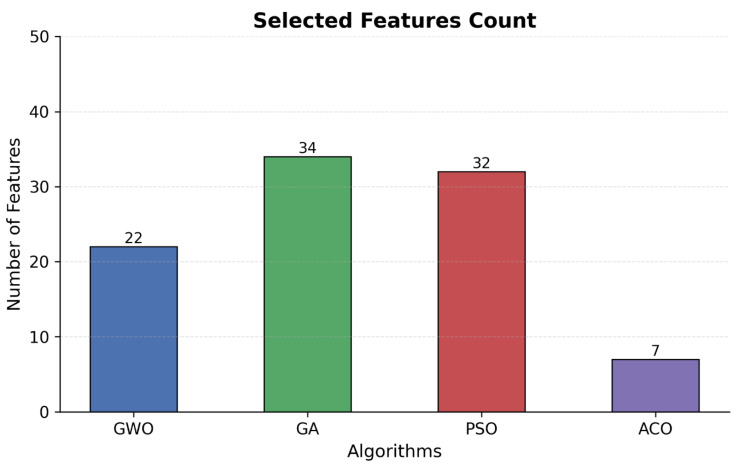
Selected features count.

**Figure 4:**
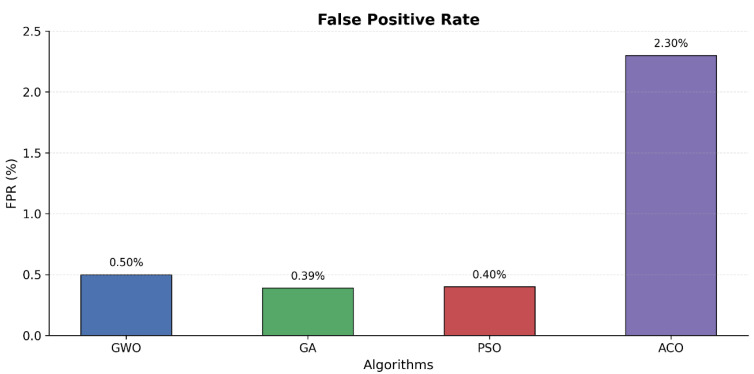
False positive rates.

**Figure 5:**
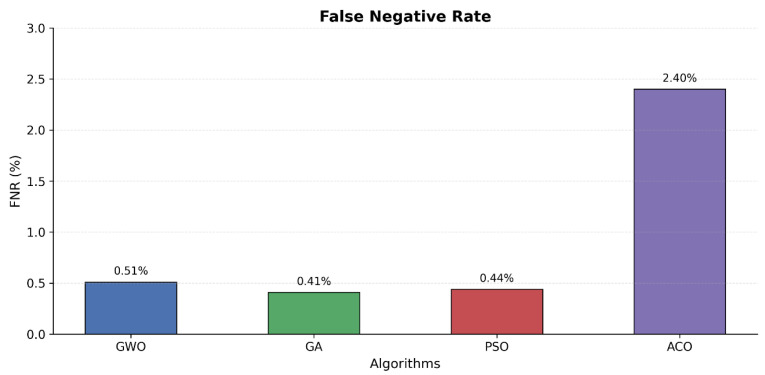
False negative rates.

Comparison of feature count presented in Figure 3 demonstrates that Particle Swarm Optimization achieved a similar level of performance at 99.58% using 32 features but achieved the overall longest processing time of 26,477 s. The error analysis presented in Figure 4 and Figure 5 indicates that Ant Colony Optimization achieved the biggest feature reduction by choosing only 7 features (88.89% reduction) and achieving the shortest processing time of 3001 s but with lower accuracy of 97.65% and high false positive and negative rates.

**Figure 6:**
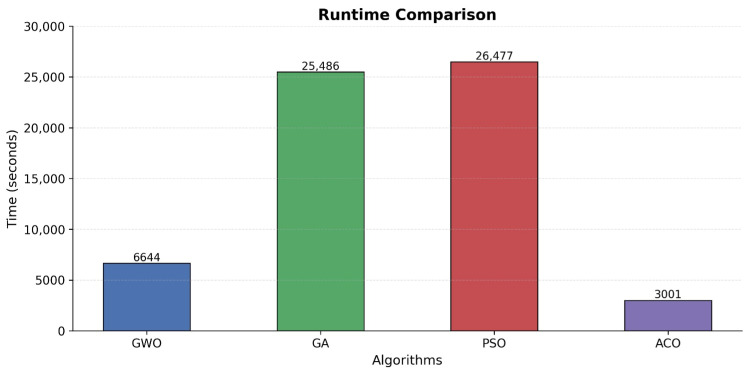
Runtime comparison.

Comparative runtime evaluation shown in Figure 6 further shows that the statistical comparison carried out using the Wilcoxon rank-sum test established significant differences (*p* < 0.05) among the algorithm performance on all the measures considered. We report exact *p*-Values and effect sizes (Cliff’s δ) for key pairwise contrasts, e.g., MOGA vs. MOGWO [*p* = 0.012, δ = 0.23] and MOPSO vs. MOACO [*p* = < 1 × 10^−4^, δ = 0.85]. Additionally, the computation of the effect size indicated large practical implications on accuracy differences of the MOACO and other algorithm pairs, and moderate effect size on the comparison of feature reduction.

### 4.2. Trade-Off Analysis and Multi-Objective Optimization Effectiveness

The comparison of runtime shown in Figure 6 confirms that a statistical analysis using the Wilcoxon rank-sum test showed significant differences (*p* < 0.05) in algorithm performances for all considered measures. The effect size computation indicated large practical significance for accuracy difference contrasts of MOACO versus the other algorithms, but moderate effect sizes for measures corresponding to feature reduction contrasts.

The comparison of trade-offs identifies three distinct groups of performance. The high-accuracy set includes MOGA, MOGWO, and MOPSO, each achieving more than 99.50% accuracy, but with varying feature requirements. In particular, MOGA and MOPSO require 34 and 32 features, respectively, but MOGWO achieves similar performance requiring just 22 features, showing a feature usage reduction of 35% compared to MOGA. The efficiency set is given by MOACO, achieving the largest feature reduction of 88.89%, but at the cost of a high accuracy loss of nearly 2%.

Pareto front analysis reveals significant differences in solution diversity and cov-erage. MOACO generated the largest number of Pareto solutions (1339), indicating extensive exploration of the objective space, followed by MOGWO (738 solutions). In contrast, MOGA produced only 10 Pareto solutions, suggesting rapid convergence to a narrow region of high-quality solutions. MOPSO maintained moderate diversity with 155 solutions, representing a balanced exploration-exploitation strategy.

Temporal analysis of the convergence behavior indicates that MOACO converges the quickest (3001s) because of its aggressive characteristic pruning approach, and MOGWO portrays constant convergence with well-balanced exploration. MOGA and MOPSO exhibit analogous convergence tendencies but use considerably more computational time because of their sophisticated population-based procedures and lengthy feature evaluation processes.

### 4.3. Practical Implications, Deployment Considerations, and Limitations

Practical deployment of multi-objective feature selection methodologies for real-world intrusion detection systems necessitates consideration of operational constraints, deployment conditions, and performance needs, and our experimental results yield insightful guidance for implementers. For enterprise net-work deployments with prolific computational capacity and stringent security needs, MOGA is the best choice, producing the best accuracy (99.60%) and lowest false positive ratio (0.39%); at 25,486 s, its lengthy training time is tolerable for offline model building, and the 34-feature set provides thorough menace coverage at bearable computational cost in online detection. For balanced performance needs, MOGWO provides the best balance of accuracy and efficiency, attaining 99.50% accuracy using merely 22 features (reducing feature set by 65%) and providing a 6644 s training time, enabling periodic model updates at the cost of real-time detection feasibility. For resource-starved IoT and edge-based deployments, MOACO offers the most expedient solution, using just 7 features (89% reduction) and featuring the quickest training times (3001 s); at the cost of reduced accuracy to 97.65% (fastest training: 3001 s; vs. 6644 s GWO, 25,486 s GA, 26,477 s PSO). It may provide adequate preliminary menace screening for bandwidth-starved or energy-starved devices. Finally, for cloud-based security-as-a-service deployments that need high throughput, MOPSO provides competitive accuracy (99.58%) using 32 features, albeit its lengthy training times of 26,477 s render it more suitable for batch processing use cases.

The 820,834-sample test verifies algorithm scalability for large deployments. The memory is contained by every algo-rithm, and MOACO makes the lowest resource consumption by virtue of having low requirements for features. The network delay analysis verifies that low delay processing of network traffic is realized by deployments having the 7-feature subset of MOACO, but due to high-throughput deployments, potential delay is induced by the 34-feature requirement of MOGA.

There are various limitations to be identified in this study. First, the evaluation relies on a solitary dataset, which, in spite of its extensiveness, might not include every variation occurring in network spaces. The binary framework of classification (normal/attack) eclipses the naturally multi-class nature of real-world intrusion detection, where the detection of designated attack types is called for. The static choice of features scheme is not able to support the dynamic nature of network patterns of traffic and the evolution of attack plans. Real-world deployments might require adaptive feature choice schemes that would be able to adjust to varying network conditions and newly arising patterns of threats. The computational analysis was carried out under laboratory conditions of control using standardized hardware configurations. Production deployments with dissimilar hardware capacities, network conditions, and simultaneous system loads may significantly affect algorithm performance and attributes of convergence. Long runtimes for MOGA/MOPSO are a practical constraint; we recommend budgeted iterations, patience-based early stopping, population downsizing, surrogate-assisted evaluation, and hardware parallelism to reduce retraining time while preserving comparative conclusions.

The noted limitations can be addressed in follow-up work as follows: (i) Algorithm scope—extend the study to additional bio-inspired and hybrid optimizers (e.g., DE/ABC/FA/CS/BA and recent hybrids) under the same protocol to test generality. (ii) External validity—evaluate across multiple datasets (e.g., modern IoT/IIoT and enterprise traces), include cross-site and temporal-drift splits, and report cross-dataset transfer. (iii) Runtime & deployment—profile on representative edge/enterprise/cloud hardware, add streaming/online feature selection and incremental retraining schedules, and report end-to-end latency envelopes. (iv) Class imbalance & rare attacks—incorporate cost-sensitive training, calibrated thresholding, and anomaly/one-class fallbacks, with per-class metrics. (v) Downstream model dependence—replicate results with multiple classifiers to quantify robustness beyond the default DecisionTree baseline. (vi) Hyper-parameter sensitivity—add ablations and automated tuning to confirm that conclusions are stable across reasonable ranges. Our evaluation does not include per-attack (multi-class) classification; we focus on binary anomaly detection to enable a fair, apples-to-apples comparison of feature-selection strategies. As follow-on work, we will extend the pipeline to hierarchical multi-class IDS on X-IIoTID, reporting per-class metrics, confusion analyses, cost-sensitive thresholds, and drift-aware updates.

Multi-objective optimization’s characteristic is the lack of a general-purpose algorithm that is best on all criteria simultaneously. This work conveys Pareto optimality’s principle, such that every algorithm represents separate points of trading-offs among mutually exclusive objectives. The absence of an algorithm best for every criterion reminds us of the necessity of scenario-specific choice guided by operational needs. Table 3 reports the per-objective rankings—accuracy, feature compactness/reduction, error rates, and runtime—offering a concise snapshot of the trade-offs among MOGWO, MOGA, MOPSO, and MOACO.

The performance comparison proves that none of the algorithms demonstrate dominance for every criterion, justifying the multi-objective framework. MOGA is best on accuracy-based criteria but needs more features and more computational hours. MOACO is best on feature reduction and speed but at the cost of less accuracy. MOGWO is best on overall balance of various objectives, and MOPSO offers competitive accuracy at moderate resource usage.

Optimal algorithm selection relies on the particular deployment situation and working constraint. In security-sensitive sites such as banks and government agencies, the use of the MOGA is preferred due to its best accuracy (99.60%) and lowest false positive level (0.39%). MOGWO is best for well-balanced corporate networks, with 99.50% accuracy and 65% feature reduction. For resource-limited IoT edge hardware, MOACO is a must due to its 89% feature reduction and quickest processing time (3001 s). Last, but not least, MOPSO is best suited for large-scale deployments in the cloud because it preserves 99.58% accuracy and has a scalable architecture.

The comparison identifies the appearance of three separate trade-off regions. The High-Accuracy Region contains MOGA, MOPSO, and MOGWO, each achieving more than 99.50% accuracy using between 22 and 34 attributes. The Efficiency Region is mostly dominated by MOACO, which attains 97.65% accuracy using just 7 attributes, showing a feature reduction of 89%, and having the fastest execution time as well. The Balanced Region, on the other hand, is illustrated by MOGWO, which offers the best balance of accuracy and efficiency and reaches 99.50% accuracy using 22 attributes.

A summary of the best performances reveals several key findings. MOGA achieved the highest accuracy at 99.60% and the lowest false positive rate at 0.39%. In terms of feature reduction, MOACO was the best, reducing the feature set to only 7 features, an 89% reduction. MOACO was also the fastest in training, with a runtime of 3001 s. MOGWO demonstrated the best balance between accuracy and features, with 99.50% accuracy using 22 features. Finally, MOACO produced the most Pareto solutions, with 1339 diverse solutions. While representative, our algorithm set is not exhaustive within bio-inspired optimization; expanding to DE, ABC, FA, CS, BA and contemporary hybrids will provide a more comprehensive view of Pareto-front diversity in IDS feature selection.

## 5. Conclusions and Future Work

This work delivered a controlled, apples-to-apples comparison of four bio-inspired, multi-objective feature selection algorithms for intrusion detection on the X-IIoTID dataset. By standardizing preprocessing, classifier, metrics, and repeat counts, we isolated the contribution of the selection strategy itself and exposed the practical trade-offs that matter at deployment time. A central takeaway is that no single method dominates all objectives simultaneously; instead, each algorithm settles on a characteristic region of the accuracy–subset–runtime design space. This validates a Pareto perspective for IDS feature selection: practitioners should choose operating points that match their constraints rather than chase a single scalar optimum.

The quantitative results are unambiguous about where each method shines. MOGA achieved the highest accuracy (99.60%) with the lowest FPR (0.39%), using 34 features and incurring a training time of 25,486 s. This profile prioritizes correctness above efficiency and suits high-assurance enterprise networks whose risk posture and infrastructure can absorb heavier compute. MOGWO offered the best accuracy–subset balance, reaching 99.50% accuracy with just 22 features (≈65% reduction) and a 6644 s training time. It is a natural default for balanced environments where strong accuracy, substantial sparsity, and moderate training cost are all desirable. MOPSO produced near-best accuracy (99.58%) with 32 features but at the highest overall runtime (26,477 s), making it appropriate for batch/offline analysis or cloud workflows where time budgets are less restrictive. Finally, MOACO yielded the smallest subset (7 features; ≈89% reduction) and the fastest training (3001 s) at 97.65% accuracy, a compelling profile for edge/IoT devices, gateways, and first-stage screening pipelines where throughput and footprint dominate.

Taken together, these outcomes translate into actionable guidance. If maximum precision and the lowest false alarms are paramount (e.g., regulated enterprise segments, SOC triage for high-impact assets), MOGA is the most appropriate choice despite a larger subset and longer training time. For general-purpose deployments that value both compactness and high accuracy, MOGWO is the most balanced recommendation and a strong starting baseline. Where computational efficiency and model sparsity are binding constraints (battery-powered sensors, bandwidth-limited links, on-device retraining), MOACO provides the leanest solution and the fastest iteration cycle. When near-ceiling accuracy is prioritized and compute is abundant (offline forensics, periodic cloud re-baselining), MOPSO is competitive even with a higher runtime.

Beyond per-algorithm prescriptions, the study underscores two broader implications for IDS design. First, feature set size is not a mere engineering nicety—it materially affects training time, memory footprint, and latency, thereby shaping where a model can be deployed (edge vs. enterprise vs. cloud) and how often it can be refreshed. Second, explicit multi-objective evaluation is essential: reporting only accuracy obscures crucial trade-offs and can lead to choices that fit a benchmark but fail in production. Our results show that a few percentage points of subset reduction or runtime improvement can be decisive in real-world pipelines, especially under streaming loads and concept drift.

While our protocol is standardized and our conclusions are robust across repeated runs, several limitations point to fruitful extensions. The algorithm set, though representative, is not exhaustive within bio-inspired optimization; DE, ABC, FA, CS, BA, whale and other recent hybrids warrant inclusion under the same protocol to test generality. External validity should be broadened by evaluating multiple modern datasets and splits that stress temporal drift and cross-site transfer. From a deployment standpoint, profiling end-to-end latency and energy on representative edge/enterprise/cloud hardware would further align conclusions with operational realities. Methodologically, hyper-parameter sensitivity and ablation studies can confirm stability of rankings under reasonable ranges, while cost-sensitive learning and calibrated thresholds can better reflect asymmetric risks across attack families.

Two promising directions follow naturally. The first is streaming/online feature selection with incremental retraining, turning multi-objective selection from a one-off batch decision into a continual process resilient to drift. The second is hybrid scheduling: for example, a MOACO-first pass for rapid pre-filtering on the edge, escalating candidate events to a MOGA-backed verifier in the cloud or data center. Such tiered designs pair the speed and sparsity of one method with the precision of another, leveraging each algorithm where it is strongest.

By framing IDS feature selection as a bi-objective problem and comparing MOGWO, MOGA, MOPSO, and MOACO under a unified, reproducible setup, we provide a clear, deployment-oriented map of the accuracy–subset–runtime landscape. Practitioners can use this map to select algorithms aligned with their constraints: MOGA for maximum precision, MOGWO for balance, MOACO for efficiency and sparsity, and MOPSO for near-best accuracy when runtime is secondary. Future work will extend coverage to additional optimizers and datasets, deepen drift-aware evaluation, and explore online and hybrid strategies that further tighten the link between multi-objective selection and operational IDS performance. These findings are dataset-specific and should be read as a baseline under a simple classifier. Broader validation on multiple datasets and stronger learners will test the portability of the selected subsets and quantify end-to-end gains.

## Figures and Tables

**Table 1:** X-IIoTID dataset summary and feature taxonomy used in our experiments.

Item	Value
Total records Total features Feature sources	820,834 (Normal: 421,417, Attack: 399,417)–51.34% Normal, 48.66% Attack
	63 (includes 3 label levels: normal vs. attack, attack sub-category, attack-sub-sub-category)
	Network traffic, system logs, application logs, device resources, commercial IDS logs
Protocols & connectivity	New IIoT protocols and industrial/transport, traffic across edge, mobile, and cloud
Attack families (examples)	Reconnaissance (scanning, fuzzing, discovery), Weaponisation (brute force, dictionary, insider), Exploitation (reverse shell, MitM), Lateral movement (MQTT broker, Modbus register read, TCP relay), C2/Data exfiltration/Tampering (false data injection), Ransomware, DoS
Label granularity	Binary (Normal/Attack) and hierarchical multi-class via sub-category & sub-sub-category labels

**Table 2:** Comparative performance of multi-objective algorithms.

Algorithm	Accuracy (%)	Selected Features	Feature Reduction (%)	FPR (%)	FNR (%)	Runtime(s)	Pareto Solutions
MOGWO	99.50	22	65.08	0.50	0.51	6644.22	738
MOGA	99.60	34	46.03	0.39	0.41	25,485.80	10
MOPSO	99.58	32	49.21	0.40	0.44	26,476.83	155
MOACO	97.65	7	88.89	2.30	2.40	3001.21	1339

**Table 3:** Algorithm ranking across different objectives.

Objective	1st Place	2nd Place	3rd Place	4th Place
Accuracy	MOGA (99.60%)	MOPSO (99.58%)	MOGWO (99.50%)	MOACO (97.65%)
Feature Reduction	MOACO (89%)	MOGWO (65%)	MOPSO (49%)	MOGA (46%)
Low FPR	MOGA (0.39%)	MOPSO (0.40%)	MOGWO (0.50%)	MOACO (2.30%)
Fast Training	MOACO (3001 s)	MOGWO (6644 s)	MOGA (25,486 s)	MOPSO (26,477 s)
Pareto Diversity	MOACO (1339)	MOGWO (738)	MOPSO (155)	MOGA (10)

## Data Availability

The research described in this paper was done using publicly available datasets. These datasets can be accessed online through the links provided by their authors in the corresponding publications.

## Abbreviations

The following abbreviations are used in this manuscript:

ACO	Ant Colony Optimization
AE	Autoencoder
CNN	Convolutional Neural Network
CPS	Cyber-Physical System
GA	Genetic Algorithm
ICS	Industrial Control System
IIoT	Industrial Internet of Things
IoT	Internet of Things
IoV	Internet of Vehicles
MOACO	Multi-Objective Ant Colony Optimization
MOGA	Multi-Objective Genetic Algorithm
MOGWO	Multi-Objective Grey Wolf Optimizer
MOPSO	Multi-Objective Particle Swarm Optimization
SDN	Software-Defined Network
XAI	Explainable Artificial Intelligence
